# Computational Methods for the Discovery of Metabolic Markers of Complex Traits

**DOI:** 10.3390/metabo9040066

**Published:** 2019-04-04

**Authors:** Michael Y. Lee, Ting Hu

**Affiliations:** 1 Faculty of Medicine, Memorial University, St. John’s, NL A1B 3V6, Canada; mylee@mun.ca; 2 Department of Computer Science, Memorial University, St. John’s, NL A1B 3X5, Canada

**Keywords:** metabolomics, complex diseases, biomarker discovery, machine learning, feature selection, classification, ensemble learning, artificial neural networks, genetic programming

## Abstract

Metabolomics uses quantitative analyses of metabolites from tissues or bodily fluids to acquire a functional readout of the physiological state. Complex diseases arise from the influence of multiple factors, such as genetics, environment and lifestyle. Since genes, RNAs and proteins converge onto the terminal downstream metabolome, metabolomics datasets offer a rich source of information in a complex and convoluted presentation. Thus, powerful computational methods capable of deciphering the effects of many upstream influences have become increasingly necessary. In this review, the workflow of metabolic marker discovery is outlined from metabolite extraction to model interpretation and validation. Additionally, current metabolomics research in various complex disease areas is examined to identify gaps and trends in the use of several statistical and computational algorithms. Then, we highlight and discuss three advanced machine-learning algorithms, specifically ensemble learning, artificial neural networks, and genetic programming, that are currently less visible, but are budding with high potential for utility in metabolomics research. With an upward trend in the use of highly-accurate, multivariate models in the metabolomics literature, diagnostic biomarker panels of complex diseases are more recently achieving accuracies approaching or exceeding traditional diagnostic procedures. This review aims to provide an overview of computational methods in metabolomics and promote the use of up-to-date machine-learning and computational methods by metabolomics researchers.

## 1. Introduction

Over the past decade, the metabolome has been deemed the final frontier for broad, biochemical databases of organismal information among the well-established fields of genomics, transcriptomics, and proteomics [1]. Metabolomics is the study of quantifying metabolites and mapping their complex interactions within this domain, which is comprised of the total set of small molecules (<1500 Da) present in cells, tissues, organs and biological fluids [2,3]. It is the final downstream component of the biochemical stages, involving genes, RNA, proteins and environmental factors, ultimately yielding phenotypic changes in an organism [2]. Since metabolism crucially involves important physiological processes that diseases often alter, metabolomics analyses can be used to detect disease-driven changes from the levels of thousands of metabolites, allowing for enhancements in current diagnostic methods and discoveries of specific, perturbed metabolic networks. The advantage of using metabolomics is therefore derived from its provision of a functional readout of the physiological state of an organism. This is because metabolites act as direct signatures of biochemical activity, whereas genes and proteins may be affected by epigenetic regulation and post-translational modifications. In other words, genomics reveals what may have occurred, whereas metabolomics reflects what certainly occurred.

Importantly, metabolomics may hold the key to tackling the challenges associated with complex diseases, which are caused by an intricate interplay between an individual’s genes, environment and lifestyle [4]. Interestingly, most diseases lie under this umbrella term, which include, but are not limited to cancer, cardiovascular disease, diabetes, arthritis, obesity and dementia. It is well known that the classical Mendelian patterns of inheritance are not observed within these illnesses. Rather, expression of certain correlational genes may increase risk of contraction, but does not guarantee incidence; instead, toxins from the environment, drugs consumed over one’s lifetime, poor diet and lack of exercise in combination with such genes would likely lead to disease onset. Therefore, researchers of complex diseases must identify methods to overcome the challenges of deciphering the quantitative influence of risk-associated genes in comparison to non-genetic factors. Metabolomics offers a solution to this by allowing the individual influences of genetics, environment and lifestyle to converge onto the metabolome as a terminal downstream domain of products. This holistic approach allows metabolomics researchers to discover biomarker signatures that capture the multiple major factors driving the complex disease. Ultimately, these panels can help to diagnose at-risk complex disease patients in the clinic and even predict onset years before symptoms arise using prodromal metabolomes [5]. In addition to the clinical benefits, it grants researchers a useful visualization of how the complex metabolic networks differ with and without disease influence. Research for metabolic marker discovery spans a fast-growing array of prevalent disease areas, such as breast cancer, osteoarthritis and Alzheimer’s [5,6,7].

Although rich quantitative datasets may contain valuable information, the extents of their utilities are limited by the appropriateness of the selected statistical and computational methods of analysis. Since these datasets contain hundreds of features, the value of an appropriate method would be derived from its ability to account not only for the effects of each metabolite in isolation, but in a multivariate manner with consideration of interaction-based effects. Thus, while recent advancements in analytical chemistry techniques, such as nuclear magnetic resonance (NMR) and mass spectrometry (MS), have made it possible to quantify hundreds of metabolites within a reasonable time frame, these techniques must be coupled with fitting statistical and computational algorithms to translate the data into a practical application in the clinic [8]. Unfortunately, the majority of metabolomics studies historically have not employed optimal methods for biomarker discovery, perhaps due to a lack of statistical and computational expertise among metabolomics researchers, which has spurred the publication of instructional and guideline-setting papers in the field [9,10,11,12]. Today, the Human Metabolome Database reports the existence of over 100,000 metabolites in the human body [13]. As analytical methods improve with regard to their discriminatory ability and efficiency, the quantifiable metabolome and its associated datasets will continue to grow, raising the relevance of powerful, heuristic computational methods to the forefront and placing a greater importance on their delineation to biological researchers.

The aims of this review are three-fold. First, we will outline a general workflow of the steps required from a biological question to metabolic marker discovery. Second, the current authors will provide an overview of current metabolomics research within prominent complex diseases to identify gaps and trends in computational methods’ use. Third, this review will discuss the benefits and costs of using different computational methods and highlight more recently-applied, promising machine-learning algorithms, including ensemble learning, artificial neural networks and genetic programming.

## 2. Metabolic Marker Discovery

**Figure 1:**
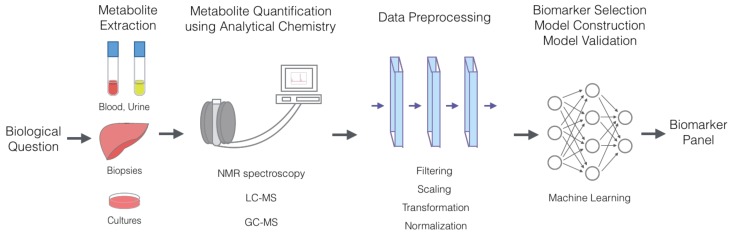
General workflow of the metabolic marker discovery process. Metabolite extraction often can be performed from cells, tissues, organs and fluids, including blood and urine. Metabolite quantification is performed using analytical chemistry techniques, such as LC-MS, GC-MS and NMR, which provide concentration values for each metabolite in solution. Biomarker selection and model construction are conducted using various machine learning algorithms.

The general workflow of metabolic marker discovery (Figure 1) typically involves forming the biological questions, extracting metabolites from cells, tissues or organs, quantifying metabolites using NMR or MS, preprocessing data to remove irrelevant biases, selecting a biomarker panel and constructing a predictive model through feature selection and classification, typically using machine learning algorithms.

There are generally two types of metabolomics studies: targeted and non-targeted. In non-targeted studies, the global metabolic profile is assessed. Thus, all detected metabolites in a sample are given the opportunity to be included in the biomarker panel. Hypotheses are not tested in this approach, but rather formed. In contrast, targeted studies focus on a selective group of metabolites to enhance specificity, precision and accuracy in testing a specific hypothesis. Furthermore, a targeted approach is useful in validating results from a global metabolic profiling (non-targeted) study [14]. It is worth noting that quantifying the data in a non-targeted manner is far from a reality due to the limitations of NMR and MS and the broad diversity of metabolite structures. However, today’s technology provides sufficient data for powerful, multivariate computational algorithms to classify people for current and future disease states with accuracies approaching or exceeding current diagnostic measures in various disease areas [5,15,16].

The two most common metabolite quantification techniques used in metabolomics are MS and NMR spectroscopy. In MS, the metabolite is ionized before analysis. Using charged ion modes, the ion signal is converted into mass spectra. By examining the resulting peaks, molecular mass is determined with the mass-to-charge ratio. There is a diversity of MS techniques that exist for complementary purposes, such as gas chromatography MS (GC-MS) and liquid chromatography MS (LC-MS). Each type takes advantage of certain physicochemical properties of the assessed metabolites to separate samples into their constituents. Metabolites with low molecular weights are analysed using GC-MS, whereas LC-MS is capable of evaluating a higher weight range. MS is able to provide quantitative data with high sensitivity and selectivity. In contrast, NMR spectroscopy employs a magnetic field to exploit angled spins and properties of atoms in the molecule of interest, which consequently absorb and re-emit electromagnetic radiation. Detectors on the NMR device use this signal to produce results that are comparably more quantifiable and reproducible and do not destroy measured samples. Furthermore, although MS can detect a broader range of metabolites, it is less time efficient in comparison to NMR; however, both methods are used to examine a combined, wider range of metabolites [14].

Data preprocessing is the general preparatory stage of the data, which ensures that the resulting dataset can be analysed without major issues. Each step of the process contributes to an overall removal of biases and incomplete features. For reference, patients in a metabolomics study belong broadly to the category of samples (or rows), whereas metabolites are classified under features (or columns). First, normalization is one critical step in this process and includes various variable types such as sample normalization, probabilistic quotient normalization, and quantile normalization [17,18]. Sample normalization simply requires tying all samples to a standard value for one particular metabolite in an attempt to resolve significant discrepancies across entire samples due to varying fluid dilution levels. Second, filtering is notably valuable for its ability to eliminate metabolites that have an excessive percentage of missing values across samples and a concentration constancy independent of group classification. A tolerable percentage of present data would typically be 80%, and the maximum relative standard deviation for constancy evaluation would be approximately 15% [9]. Regarding transformation, it is common to observe replacement of all values with their logarithmic outputs (i.e., *x* becomes log(x)). The logarithmic transformation improves the normality of the data distribution, since metabolomics data have been shown to mostly follow a log-normal distribution [17]. This also conveniently provides the benefit of working with values within a much narrower range, improving pattern visualization and interpretability for datasets containing extremely large values. Finally, scaling provides a way to address large differences in metabolite levels across patients through standardization down each feature. Typically, the sample mean is subtracted from each data point and divided by the sample standard deviation, removing potential biases related to absolute quantities [9]. Upon completion of the appropriate preprocessing methods, metabolomics researchers may proceed to statistical and computational analysis.

Population-based metabolomics looks for metabolic markers that can provide the best discriminating power between the diseased cases and healthy controls. These metabolic markers in turn can help us develop highly-cost-efficient and effective drugs that target enzymes involved in key processes for better disease treatments, as well as construct a computational model to predict the clinical outcome for new patients [9,19,20,21]. Biomarker discovery in omics science usually follows a three-step scheme. In the following text, we discuss the objectives, most commonly-used methodologies and the challenges of each step.

The first step is biomarker selection, or attribute selection, where only the most relevant bio-attributes are identified. The necessity of attribute selection is due to the high-dimensionality of most omics data, where hundreds to a million attributes can be considered for their potential association with diseases. Removing irrelevant attributes reduces the computational overhead of downstream analyses, simplifies the learned model and guides the search for biomarkers since the true signal in the data will be more predominant after the noise is removed [22,23].

For attribute selection in omics, most existing studies use univariate tests, such as Wilcoxon, Kruskal-Wallis tests, or additive multivariate analyses, such as logistic regression, least squares regression (LSR) or discriminant analysis. Attributes are examined separately or combined additively for their association with the disease outcome, and only those with significant main effects are usually selected. Such analyses inherently overlook the synergistic non-linear interactions among multiple attributes. Given the complexity of human diseases, it is more plausible that multiple factors interact synergistically, and one factor’s effect on the disease depends on others’. However, looking for combinations of attributes exposes us to an enormous search space since the total number of all possible combinations with all orders for *n* attributes is $2^{n}$. Even a small number of n=10 translates to 1023 subsets of attributes to be tested. For n=1000, enumerating all combinations of only the orders of two and three (i.e., pairwise and three-way synergy) requires 499,500 and 166,167,000 tests, respectively. If we used all the computers currently known on this planet, it would still be impossible to exhaustively enumerate and test all possible combinations of attributes of all orders by a meaningful deadline. Therefore, powerful heuristic search algorithms are needed [24,25].

The second step is model construction. This step uses identified important biomarkers to construct a classification model that can predict a new subject with a high or low risk of developing the disease. Model construction is usually carried out using machine-learning algorithms through a training process on population-based omics data [9]. Although biomarker selection and model construction depend on each other since only the selection of the most relevant attributes can yield an accurate and general prediction model, they are usually done separately. A filter method is usually employed to select the attributes, and the subsequent classification algorithm for model construction is independent of the filter method. These two steps can also be wrapped in an iterative process to further refine their results. That is, a heuristic search algorithm provides a subset of attributes, and model construction trains a model and feeds back to attribute selection in the next round. However, such an iterative method imposes a large computational cost due to repetition.

The third step in biomarker discovery is biological interpretation and validation of the discovered biomarkers and the constructed predictive model. This is particularly challenging since many machine learning algorithms produce a “black box” model, which can be uninformative for interpretation and validation [26,27]. In many other data-rich fields, such as information technology and finance, prediction accuracy alone may often be sufficient for decision making. However, model interpretation would be particularly important in bioinformatics since the biological mechanisms underlying the model must be understood for us to transfer knowledge to clinical applications.

Model validation in metabolomics typically involves a random splitting of metabolomics samples into 80% training and 20% test sets; however, this ratio may depend on the number of patients and metabolites available for analysis. Furthermore, cross-validation is a useful technique that repeats this process multiple times with different training and test sets, ultimately utilizing the average of the evaluated model validity measures. Additionally, it is important to be aware of the risk of achieving a local optimum rather than the global, but the loss value of such an event may depend on the complexity of the generated model (particularly in artificial neural networks) and may be mitigated with cross-validation, ensemble learning and population-based model search [28]. Feature selection and model construction use training datasets, and the evaluation of a predictive model should always be reported using the unseen test set. This ensures the generalization of the trained model that can translate to future incoming data. In addition to statistical validation, for bioinformatics research on metabolic marker discovery, it is crucial to use independent data in order to replicate the findings and to include follow-up biological experiments to further validate the mechanistic hypotheses generated by the informatics studies.

## 3. Current Research in Metabolomics, Complex Diseases and Computational Methods

The current literature regarding the investigation of complex diseases using computational methods in metabolomics is rapidly growing. Metabolites are small endogenous or exogenous molecules that play a direct role in energy homoeostasis, macromolecule synthesis, waste elimination and biological regulation [2,3]. These molecules exist in cells, tissues, organs and fluids, including cerebrospinal fluid (CSF), blood and urine. Methods of metabolite extraction are especially important for future clinical applications of metabolomics since safety and cost are likely to influence the adoption of a new diagnostic test. Metabolomics offers a useful new entry in the world of diagnostics, as acquiring blood or urine samples is minimally invasive to non-invasive. Furthermore, metabolomics has a unique capacity to provide insights into an individual’s physiological state and capture the multi-causal nature of a complex disease. Today, the majority of complex disease research has shifted from univariate and additive multivariate techniques to newer, more powerful multivariate methods. This review sets its focus on Alzheimer’s, breast cancer and osteoarthritis, as complex diseases with substantial metabolomics research history and recent advancements in the application of computational methods to the disease area.

### 3.1. Alzheimer’s Disease

Alzheimer’s disease (AD) is a neurodegenerative disorder that is characterized by progressive cerebral atrophy and hypometabolism [29]. Clinical symptoms include memory deficits, language challenges and personality changes. The AD population in the U.S. is projected to triple by mid-century, highlighting the urgency and utility of having concrete developments toward an effective treatment or cure [30]. Currently, amyloid beta (Aβ) plaques and neurofibrillary tau tangles are believed to be the primary neuropathological substrates contributing to pathogenesis [31]. This leads to a slow buildup of plaques and tangles over the course of the prodromal (20 years) and clinical phases (8–10 years), ultimately ending in mortality [31]. However, recent clinical trials that have successfully cleared Aβ plaques from AD brains have failed to demonstrate symptomatic improvements [32]. With only four non-curative FDA-approved drugs and an abysmal failure rate for clinical trials (i.e., 99.6%), efforts to investigate the early disease stages and develop novel diagnostic measures for preclinical prevention and management have become increasingly present and valued in the field [33].

One early study in AD metabolomics revealed that sulfatide species (a class of myelin-specific sphingolipids) were reduced significantly in brain tissue lipid extracts from patients with mild AD [34]. Furthermore, ceramide levels were discovered to be increased three-fold in white matter. These findings were determined using linear correlations and analysis of variance (ANOVA), which provided enough information to support that there was an associative relationship between one particular class of species and dementia severity score, but did not account for the combined effect of multiple metabolites. Years later, Han et al. showed that it was possible to achieve a similar finding from a blood sample quantification of over 800 different lipid species using shotgun lipidomics [35]. A metabolic signature was formed using this “broad-stroke” method of metabolomics study. This analysis revealed the value of analysing large datasets to establish a metabolic signature. Yet, Wilcoxon rank sum tests, a univariate method, were used to uncover the significant differences between the AD and control groups amongst the complex combinatorial and interactive possibilities of these hundreds of metabolites.

Wang et al., addressed this statistical limitation in their study, which examined a relatively large cohort of 172 individuals with an additional third group of mild cognitive impairment (MCI; considered to be an early form of AD) patients [36]. The researchers quantified the concentrations of 238 small-molecule core metabolites (including fatty acids, amino acids, nucleic acids and carbohydrates) from plasma samples and subsequently employed several machine-learning methods for multivariate analyses, such as logistic regression, principal components analysis (PCA) and partial least-squares-discrimination analysis (PLS-DA) to determine that six metabolites, including arachidonic acid, *N*,*N*-dimethylglycine, thymine, glutamine, glutamic acid and cytidine, accounted for the differences between AD patients and controls. A similar analysis was performed for the MCI group against the healthy controls, unveiling five important metabolites, three of which were shared for the AD comparison. The area under the curve (AUC), a measure of classifier performance, yielded high scores of one and 0.998 in a training set, for the AD and MCI groups against controls, respectively. This study demonstrated the utility of an analysis that could capture the multi-factorial nature of AD within a single computational model, leading to a more accurate biomarker panel.

Most recently, Varma et al. analysed serum samples from two longitudinal cohorts of 767 prodromal individuals from the Alzheimer’s Disease Neuroimaging Initiative (ADNI) and 207 preclinical AD samples from the Baltimore Longitudinal Study of Aging (BLSA) [5]. Two machine-learning methods, support vector machine (SVM) and random forest (RF), were used to identify a 26-metabolite panel that performed with 83.33% accuracy, 86.67% sensitivity and 80% specificity. The longitudinal nature of this study allowed for an evaluation of a host of metabolite correlates with MRI measures of brain atrophy, AD pathology (i.e., Aβ concentrations in CSF), conversion risk to incident AD and cognitive performance over time. Enhanced blood levels of sphingolipid species were found to be correlated with post-mortem AD pathological severity and preclinical disease progression. Importantly, the uncovered 26-metabolites were involved in various AD-related pathways, including tau phosphorylation, Aβ metabolism, acetylcholine biosynthesis and apoptosis. This was a multi-centre study with regard to collection and analysis, which allowed for the acquisition of the largest sample size to date for an AD metabolomics study. The use of SVM and RF, which are superior multivariate methods to PLS-DA and other additive algorithms when working with large, highly-complex datasets, allowed these authors to extract more accurate information from the ADNI and BLSA data [5].

Overall, AD research is trending in the direction of using computationally-robust, multivariate methods to develop new diagnostic tests that are safer, more cost-efficient, and have similar or greater accuracy rates than current neuropsychological and imaging techniques (which are estimated at 77%) [37].

### 3.2. Breast Cancer

Although cancer incidence in the U.S. has declined since 1991 by 26%, it continues to be one of the 10 leading causes of death with an estimate of over 600,000 in 2018 [38]. Early detection of cancer continues to be a viable prevention strategy, but has several issues. One particular problem is that researchers must grapple with cancer’s paradoxical nature to occur commonly across a lifetime, but rarely present itself at a single point in time, which magnifies the challenge of achieving acceptable test performance [39]. Current biomarkers, such as prostate-specific antigen (PSA) and carcinoembryonic antigen (CEA), have been less useful than expected either due to a low positive predictive value (PPV) or lack of survival benefit [40,41]. With the complex array of contributors to cancer, it would be prudent to consider a multivariate method for screening and diagnostic purposes.

Across cancers, metabolism, the intricate collection of intertwined pathways of energy substrates and enzymatic regulation, is dramatically altered. The central shift involves a phenomenon termed the Warburg effect, which represents the change toward the use of aerobic glycolysis to generate adenosine 5′-triphosphate (ATP) and lactate, even though it is less efficient than oxidative phosphorylation [42]. However, beyond this cellular shift, the hallmarks of cancer, including selective growth and proliferative advantage, altered stress response favouring overall survival, vascularization, invasion and metastasis, metabolic rewiring, an abetting microenvironment and immune modulation, reveal a deeply physiological nature to the disease regardless of the cancer’s origin [43]. With metabolomics at the forefront of data-driven physiological research, the field has been an important area of research for cancer with notable developments in recent years.

In particular, breast cancer is the source of 25% of all cancer cases and causes over 500,000 deaths annually [44]. Survival rates depend greatly on early detection, which is often times expensive with imaging methods, such as mammography and magnetic resonance imaging (MRI) [45]. Furthermore, mammography has been shown to miss approximately 15% of breast masses, and surgical biopsies are necessary to confirm definitively the malignancy of the tissue [46]. Testing for metabolic markers of breast cancer may allow for the development of less expensive, less invasive, and more accurate diagnostic techniques.

Similar to AD, cancer research began with univariate statistical analyses during its early years of biomarker discovery. Several studies used such techniques to infer biomarker significance, examining only a handful of metabolites [47]. Furthermore, most of these studies do not provide values supporting the utility of the biomarker, such as sensitivity, specificity and AUC [9]. As analytical chemistry techniques have improved in scalability and efficiency over the years, more studies have begun to utilize multivariate methods of analysis. In 2011, Hilvo et al. used ultra-performance LC-MS to assess the lipids in normal breast tissues compared to those that were cancerous [48]. In particular, the kernel-based orthogonal projections to latent structures (K-OPLS) method was used to generate a predictive model for estrogen receptor (ER) status based on altered lipid concentrations. Furthermore, a validation cohort was used showing similar results. The AUC was found to be 0.94 and 0.88 in the training and validation sets, respectively. In 2015, Huang et al. utilized GC-MS to profile serum samples from patients with malignant and benign breast tumour, as well as healthy controls. They then employed random forests (RF), a machine-learning multivariate technique, to assess the quality of their identified relevant serum metabolites [49]. The prediction accuracy, sensitivity and specificity between malignant breast cancer patients and healthy controls were 100%, 97% and 98%, demonstrating the assessed metabolites’ high performance as predictors.

Perhaps one of the most computationally-forward-looking studies to date has been a recent study published in 2018 on using deep learning, a subset of the neural network category of machine-learning methods, to predict accurately estrogen receptor status in breast cancer samples [6]. In the study, feed-forward networks, a framework utilizing deep learning, was compared to other machine learning techniques, including RF, SVM, prediction analysis for microarrays (PAM), generalized boosted models (GBM), recursive partitioning and regression trees (RPART) and linear discriminant analysis (LDA), with data from 162 metabolites. The results demonstrated that deep learning with its AUC of 0.93 was superior to all other tested methods. Moreover, they were able to uncover eight pathways involved in breast cancer, including central carbon and glutathione metabolism, which were not revealed from the other analyses. Overall, these researchers showed that deep learning has utility within the scope of medium-sized databases and can offer network knowledge between metabolites that other machine learning techniques may lack the capability to provide.

Across research studies for breast cancer, there appears to be evidence of a shift toward more computationally-powerful tools to understand large, complex cancer metabolomics databases. As one of the primary recommendations of the current review, this shift is necessary to improve our current biomarker panels and adapt to the rapidly-increasing number of metabolites and patients available for analysis. Utilizing the latest algorithms, such as feed-forward networks and select heuristic techniques, will promote the development of more representative models with greater potential for translation to practical, clinical applications. In a field like cancer where early diagnosis may mean the difference between a slim one-year survival rate and a simple surgical resection, advancements in metabolomics and subsequent knowledge translation efforts in diagnostics will contribute notable differences to the lives of those soon to be afflicted.

### 3.3. Osteoarthritis

Among the global population of individuals over the age of 60, osteoarthritis, a degenerative disease of joint cartilage and underlying bone, has a notable prevalence of 10% [50]. It is the most common type of arthritis and the leading form of disability in developed countries [51]. In particular, knee osteoarthritis, which accounts for over 80% of the disease burden being the primary cause of mobility-based disability, has doubled in prevalence in three generations [52]. Molecular theories explaining the pathogenesis of osteoarthritis primarily involve the aging process in association with inflammation, senescence, mitochondrial dysfunction and oxidative stress and changes in energy metabolism and cell signalling [53]. Other important predictors include old age, female sex, overweight status and obesity, muscle weakness, knee injury, frequent joint use, bone density and possibly dietary factors [54]. Therefore, metabolomics can play an important role in quantifying the multi-faceted character of osteoarthritis, especially since the combination of genetic markers and epidemiological factors, such as age, sex and BMI, has been shown to produce a relatively low AUC (i.e., 0.668) [55].

In 2014, Zhang et al. conducted a seminal study on the classification of osteoarthritis into subtypes based on metabolomics data [56]. This study used synovial fluid samples in an effort to enhance the connection between the analysed metabolome and physiological reality. With 80 osteoarthritis patients, the researchers employed PCA, cluster analysis and PLS-DA to perform a multivariate analysis on 168 quantified metabolites. These methods yielded results demonstrating that osteoarthritis was actually composed of two distinct groups, as a result of differences in the levels of 86 unique metabolites. This novel finding provides a deeper understanding of the disease phenotype, which could be further investigated in physiological and cellular studies to identify differences in molecular targets and ultimately improve drug specificity. The lack of curative drugs for the disease underscores this need for more knowledge-building, data-driven investigations in contrast to drug development efforts.

Another study examined knee osteoarthritis, developing global serum profiles for 60 individuals (including a control group) using a dataset of 106 metabolites [57]. With PCA and PLS-DA, the researchers identified a 14-metabolite signature, involved in the metabolism of energy, purines, amino acids, fatty acids, lipids and glycolysis. It was found to have an accuracy measure of 0.662. One limitation of the study was the use of serum as opposed to synovial fluid, which may have provided improved sensitivity and specificity values. However, providing evidence for the use of serum may be beneficial since it has safety and cost benefits over other fluids, despite its potential shortcomings in accurately reflecting the products of the disease process.

In 2018, Hu et al. demonstrated the utility of genetic programming (GP), a heuristic multivariate evolutionary machine-learning technique, in osteoarthritis metabolic marker discovery [7,58]. The authors applied the process of evolution on computer models, generating hundreds of potential models, selecting for the best-performing ones, breeding them together to produce children and repeating the process. Iterating through hundreds of generations with a dataset of 389 samples and 167 metabolites ultimately led to the discovery of nine key metabolites, specifically arginine, C16, C18:1, isoleucine, nitrotyrosine, ornithine, taurine, threonine and tyrosine, several of which had not been reported previously in the literature. Furthermore, genetic programming was found to perform more highly than logistic regression (a non-heuristic method) with AUC values of one and 0.91, respectively.

Overall, similar to other complex disease areas, osteoarthritis appears to be trending toward the use of multivariate, heuristic approaches that are apt at generating high-performing predictive models for the endless ways in which the upstream pathways of genes, RNA and proteins may converge.

## 4. Advanced Learning Methods for Metabolic Marker Discovery

The previous section delineated the use of more recent and advanced machine-learning methods, revealing significant improvements on metabolic marker selection and predictive model construction as a result. Table 1 summarizes the most commonly-applied classification algorithms for metabolic marker discovery, as well as a set of less utilized, but potentially powerful methods that we will spend more length explaining in this section.

Logistic regression (LR), partial least squares-discriminant analysis (PLS-DA) and recently support vector machine (SVM) are among the most currently-used statistical tools for metabolic marker discovery and predictive model training. This is likely a result of the extensive methodological research on these methods and the abundant availability of analysis packages in various programming languages including R and Python. The curation of large-volume, high-dimensional big data across multiple disciplines has been driving the methodological development of machine-learning algorithms [66,67]. Recent advanced learning algorithms have seen increasing applications to computer vision, natural language processing, pattern recognition, social sciences and medicine. Their potential has not been fully explored in the youngest member of omics, metabolomics, but they are very naturally suited to tackling the metabolic marker discovery task, which can be easily formulated as a typical feature selection and classification problem in machine learning. Here, we introduce three types of advanced learning algorithms, including ensemble learning, artificial neural networks, and genetic programming, and discuss their potential applications for metabolic marker discovery.

### 4.1. Ensemble Learning

In more complex datasets, the trained predictive models are often found to have high instability, a phenomena characterized by Breiman [68], where many distinct models involving different feature subsets can achieve comparably good training or testing prediction accuracy. Ensemble learning was proposed to address the issue by aggregating over a large set of competing base learners. Base learners are predictive models trained separately or sequentially and are often weighted based on their prediction performance. The final prediction is thus decided through majority voting for classification and averaging for regression tasks.

Base learners are usually generated from training data by one or multiple learning algorithms, resulting in a homogeneous or a heterogeneous ensemble. The learning algorithms can be any classification or regression algorithm. In the most common ensemble learning algorithms, a homogeneous ensemble is comprised of diverse classification and regression trees (CART) [69,70]. CART typically use internal nodes to represent features and use the best feature value cut-offs to spit samples into branches to reach leaf nodes representing the class labels (for classification) or target variable (for regression).

There are different mechanisms that can effectively construct the ensemble of base learners. The most commonly-used ones are bagging and boosting. Bagging is short for bootstrap aggregating and uses bootstrapped samples of the training data to train independent decision trees [69]. A bootstrapped training set is obtained by randomly sampling the training data with replacement. Therefore, a training sample may have multiple copies or not be present in a bootstrapped training set. Each bootstrapped training set is used independently to derive one decision tree. Bagging then decides the final prediction/regression outcome by majority voting or averaging these decision trees. The random forests (RF) algorithm is the most well-known ensemble learning method that employs bagging.

Boosting, on the other hand, constructs an ensemble of base learners by deriving a new learner through improving the previous one in a sequential fashion [71,72]. Boosting in fact refers to a class of such iterative ensemble techniques, among which gradient boosting machine (GBM) is a very popular and powerful boosting algorithm [73]. In GBM, at iteration *i*, a new decision tree approximation $F_{i}$ is derived by adjusting the decision tree approximation $F_{i-1}$ using the gradient of the loss function $\nabla L(y,F_{i-1})$, where *y* is the expected outcome.

Ensemble learning has been reported to have stronger generalization abilities in comparison to other machine-learning algorithms that use single predictive models [70]. The search for a single optimal model might be imperfect especially for complex, noisy and incomplete training data, and thus, using multiple separately trained or sequentially evolved models may give a good approximation of the true nature of the data. Ensemble learning has seen increasing applications to a variety of machine learning problems and could be a powerful analysis tool for metabolic marker discovery given the complexity, high-dimensionality and incompleteness of metabolomic data.

### 4.2. Artificial Neural Networks

Artificial neural networks (ANN) refer to a collection of learning algorithms inspired by the nervous systems and the human and animal brain. They loosely mimic how a large number of neurons process information and communicate in a highly-parallel style [74,75]. Each neuron is a computing unit that takes inputs (dendrites) from other neurons, and is “activated” if the aggregative inputs reach a certain condition. An activated neuron sends information (activation signals) to others through the connection (synapses) between its output (axon) and other neurons’ inputs.

In a typical ANN, neurons are organized into several layers with the first being the input and last being the output layer. The input layer includes neurons that take explanatory inputs (e.g., metabolite concentrations), and the output layer gives one or multiple prediction outcome (e.g., the disease risk). The intermediate layers are called “hidden layers” that do not interact directly with the “environment” (i.e., either input or output) and are used to construct complex relationships that combine input variables to compute the outcome. In general, more hidden layers lead to more complexity and more accurate modelling [67].

ANNs are represented by directed graphs where each node denotes a neuron and directed edges connecting neurons representing possible communication of activation signals. ANNs typically compute using a feed-forward mechanism, where neurons of a certain layer take outputs of the predecessor layer, compute the aggregative signals and use the activation functions to generate their own outputs that will be sent to the successor layer. ANNs are usually initialized randomly and then trained using an error-back-propagation mechanism. The current classification/regression error of an ANN is calculated as the absolute discrepancy of the expected and the computed outcome. The parameters of the ANN are then updated starting from the output layer to each predecessor layer based on the gradient of the cost function.

ANNs have seen much interest in research and applications in the past decades given their superior abilities of highly-accurate function approximations [76,77]. They have been exceptionally successful in tackling complex learning tasks in computer vision, natural language processing and recently recommender systems. There is a variety of network structures proposed in order to suit various learning problems, including convolutional neural networks [78], Boltzmann machine networks [79] and generative adversarial networks [80], just to name a few.

ANNs, especially deep ANNs that employ multiple hidden layers, can be powerful learning tools to construct highly-accurate predictive models for metabolomic research on complex diseases. However, they are often regarded as less “visible”, or more difficult to interpret, especially for bioinformatics research, in terms of extracting mechanistic explanations from the learned complex models [27]. Research on designing ANN structures that are more amenable for mechanistic explanations is thus needed for a better utilization of this powerful and advanced machine-learning algorithm.

### 4.3. Genetic Programming

Many well-known machine-learning algorithms gradually adjust predictive models using the gradient of the cost function, typically defined as the prediction error (i.e., the distance of the expected and actual output of a model). Genetic programming (GP) improves randomly-generated predictive models using mechanisms borrowed from natural evolution. GP is located at the intersection of machine learning and evolutionary computing and is a lesser known, but potentially powerful algorithm for complex and incomplete modelling problems.

Evolutionary algorithms define a collection of meta-heuristic optimization and modelling algorithms inspired by natural evolution [81,82,83], and have been applied to bioinformatics on various optimization and modelling problems [23,24,84,85,86,87,88,89,90]. Evolutionary algorithms employ the trial-and-error problem-solving strategy and borrow ideas from how living organisms adapt through evolution. Various branches of evolutionary algorithms have been developed over the past decades, and they encode the solution to a problem differently. Specifically, genetic algorithm (GA) and evolution strategy (ES) solve optimization problems and typically represent candidate solutions using binary strings or real-valued vectors. As a machine-learning algorithm, GP solves modelling problems whose evolutionary individuals are regression or classification models, typically represented using expression trees or imperative programs [91,92].

**Figure 2:**
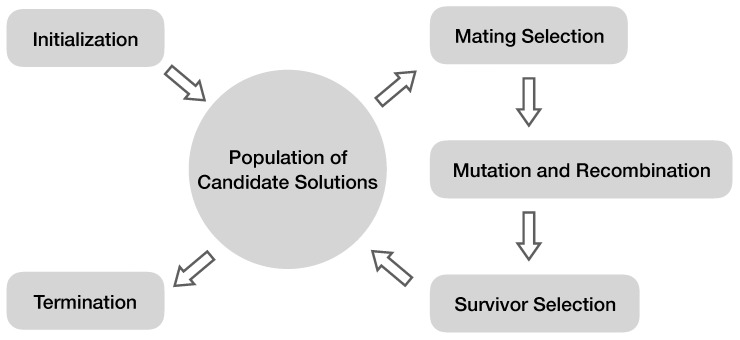
General workflow of an evolutionary algorithm. Typically, a population of candidate solutions to a problem is randomly initialized. Then, through the iterative evolution process, fitter solutions are more likely to be picked for reproduction and survival. Similar to living systems, random changes can be introduced to reproduction, such as mutation and recombination. The algorithm terminates once satisfactory solutions are observed or the computational limit (e.g., the maximal number of generations) has been reached.

Figure 2 shows the general workflow of evolutionary algorithms. An evolutionary algorithm maintains a population of diverse candidate solutions, or individuals, typically initialized randomly. These candidate solutions are compared to the desired outcome, and a fitness value can be calculated for each candidate solution based on how close it is to the desired outcome. Fitter candidate solutions will have higher probabilities of being selected for reproduction. During the reproduction process, slight and stochastic changes are applied to parent solutions, defined as mutations. Parents also swap portions of their encodings to form related, but distinctive offspring, defined as recombination. Fitter candidates survive to the next generation, and less fit ones die out. Then, through multiple generations of selection, variation and reproduction, the selection criterion (the relative distance from the desired outcome) leads the population to produce increasingly fitter solutions.

In GP, candidate solutions are symbolic models, taking the form of a syntax tree (tree GP) or a symbolic computer program (linear GP) [82,92] that map the input variables to the output. Therefore, the fitness can be naturally characterized as the prediction accuracy of such a symbolic model. Mutations can be the alteration of elements of a symbolic model, and recombination swaps sections of two symbolic models in the hope of producing better child models. In the context of metabolic marker discovery, a candidate classification model of GP takes a set of input metabolite concentration values and outputs a prediction score of the disease risk.

GP can be a powerful addition to the metabolic marker discovery toolbox. Using arithmetic and branching operators to construct predictive models allows GP to approximate highly non-linear relationships that map the input metabolite concentrations to the disease risk assessment. Moreover, given the stochastic nature of evolution, metabolic feature selection is embedded and is coevolved as the model construction in the GP algorithm. Due to the symbolic forms, the evolved predictive models are also more amenable for interpretation, in comparison to “black-box” models trained by many machine-learning algorithms.

## 5. Conclusions

Metabolomics has an incredible amount of potential in human disease studies since today’s most prominent diseases, including arthritis, diabetes, cardiovascular disease, obesity and Alzheimer’s, have clear metabolic causes [93,94,95,96]. With rapidly-developing biological, analytical and computational technologies, the concentrations of hundreds of metabolites in a biological sample can be detected within minutes [97]. The comparison of their concentration levels in phenotypically-distinguished populations (e.g., diseased and healthy subjects) can help identify pathways and biological processes associated with a certain disease. This review fulfils three primary aims. First, a delineation of the general workflow of a metabolomics study from a biological question to model validation was provided. Following this, an overview of the historically- and currently-utilized computational methods for metabolic marker discovery across prominent complex diseases, such as AD, breast cancer and osteoarthritis, were discussed for the purpose of identifying notable trends in computational technique use. Lastly, three rising areas of machine-learning methods, including ensemble learning, ANN and GP, were provided an introductory description and discussion regarding various advantages and disadvantages of usage.

Overall, there has been a clear shift in computational methodologies used by metabolomics researchers across complex disease areas. From univariate to multivariate analyses and linear to non-linear relationship modelling, the field of metabolomics is rapidly adopting the use of up-to-date machine learning algorithms to more appropriately match the intricate interplay of genetic, environmental and lifestyle factors, which converge on an estimated 100,000 downstream metabolites in the human body, in an attempt to better understand existing signalling theories and reduce significant pathway knowledge gaps that may be contributing to our lack of curative drugs for nearly all complex diseases. In the field of machine learning, improvements and innovations to these computational methods are published frequently. The importance of the choice of computational method should not be understated, as it can lead to dramatic improvements in biomarker panel performance in the clinic [6,7]. Lower performance can mean higher rates of false positives and negatives, leading to burdensome costs against the healthcare system and ultimately resulting in a reluctant phasing out of the test [40,41]. Having an understanding of existing statistical techniques, as well as new and upcoming computational methods to optimize the formation of accurate metabolic marker panels will be critical for knowledge translation efforts down the road. Therefore, this review hopes to provide researchers with an introduction to various methods for metabolomics research to advance the use of newer, potentially rich computational methods.

## Figures and Tables

**Table 1:** Machine-learning algorithms and their example applications to metabolic marker discovery.

Algorithm	Description	Examples
Logistic regression (LR)	Use a logistic function to fit a regression model for categorical outcome prediction.	[59]
Partial least squares-discriminant analysis (PLS-DA)	Find a linear subspace of high-dimensional explanatory variables to maximize the covariance between the input variables and the class label.	[60]
Support vector machine (SVM)	Use various similarity measures of training samples (also known as kernel functions) to perform linear or non-linear separation of two classes.	[61]
Random forest (RF)	Construct an ensemble of decision trees to classify training samples, as well as to assess the variable importance in the classification.	[25]
Gradient boosting machine (GBM)	Build an ensemble of decision trees in a step-wise fashion using boosting and gradient descent algorithms.	[62]
Artificial neural network (ANN)	Construct multi-layered networks of neurons to learn highly non-linear functions that map the explanatory variables to the class label.	[63,64]
Genetic programming (GP)	Use natural evolution mechanisms to automatically search for the most relevant features and classification models.	[7,65]

## Abbreviations

The following abbreviations are used in this manuscript:

NMR	nuclear magnetic resonance
MS	mass spectrometry
LC-MS	liquid chromatography-mass spectrometry
PLS-DA	partial least squares-discriminant analysis
K-OPLS	kernel-based orthogonal projections to latent structures
PCA	principal component analysis
SVM	support vector machine
RF	random forest
GBM	gradient boosting machine
ANN	artificial neural networks
AUC	area under the curve
ANOVA	analysis of variance
A*β*	amyloid beta
AD	Alzheimer’s disease
MCI	mild cognitive impairment
ADNI	Alzheimer’s Disease Neuroimaging Initiative
BLSA	Baltimore Longitudinal Study of Aging
CSF	cerebrospinal fluid
GA	genetic algorithm
ES	evolution strategy
EA	evolutionary algorithm
GP	genetic programming
ATP	adenosine 5′-triphosphate
